# Meta-analysis of Unguided Deescalation of Dual Antiplatelet Therapy in Patients with Acute Coronary Syndrome Undergoing Percutaneous Coronary Intervention

**DOI:** 10.1055/a-1827-8128

**Published:** 2022-06-13

**Authors:** Mohamed M. G. Mohamed, Safia Shaikh, Mohammed Osman, Babikir Kheiri

**Affiliations:** 1 Internal Medicine Department, SSM Health St. Mary's Hospital, St. Louis, Missouri, United States; 2 Knight Cardiovascular Institute, Oregon Health and Science University, Portland, Oregon, United States

Dual antiplatelet therapy (DAPT) is a corner stone in the treatment of patients with acute coronary syndrome (ACS) undergoing percutaneous coronary intervention (PCI). Current guidelines recommend potent P2Y _12_ inhibitors (ticagrelor and prasugrel) over clopidogrel due to their high efficacy in reducing ischemic events. 1 Nevertheless, this effect is at the expense of more bleeding risks and high expenses. 2 Different strategies have been investigated for DAPT deescalation. 2 3 Guided deescalation strategies might provide precise informed decision but conflicting data exist, and the complexities of testing and protocols might be impractical in clinical practice. 3 4 Therefore, we conducted a meta-analysis of randomized clinical trials to investigate the unguided deescalation of DAPT in ACS patients undergoing PCI.

We systematically searched multiple databases using prespecified search terms. Randomized controlled trials (RCTs) comparing DAPT deescalation after 1 month to clopidogrel versus continuation of potent P2Y _12_ inhibitors in patients with ACS undergoing PCI were included. RCTs using guided methods for deescalation were excluded. The primary outcome of interest was the net clinical benefit defined as composite of cardiovascular death, strokes, revascularization, all ischemic events, and major bleeding. Secondary outcomes included the components of the composite primary outcome. We reported odds ratio (OR) with 95% confidence intervals (CIs). Data were pooled using random effects model with RevMan 5.4 software.

Three RCTS with a total of 3,474 patients (deescalation = 1,737 and continuation = 1,737) were included. 4 5 6 The mean age was 60.3 ± 11.2 years and 82.7% were males. Patients with hypertension, dyslipidemia, and diabetes represented 49.5, 43.3, and 27.7%, respectively. The median follow-up duration was 12 months. Patients with ST-segment elevation myocardial infarction (STEMI) represented 50.1 and 25.5% of patients who had >1 vessel intervention. The average stent diameter and length was 3.1 ± 0.5 and 29 ± 14 mm, respectively.

**Fig. 1:**
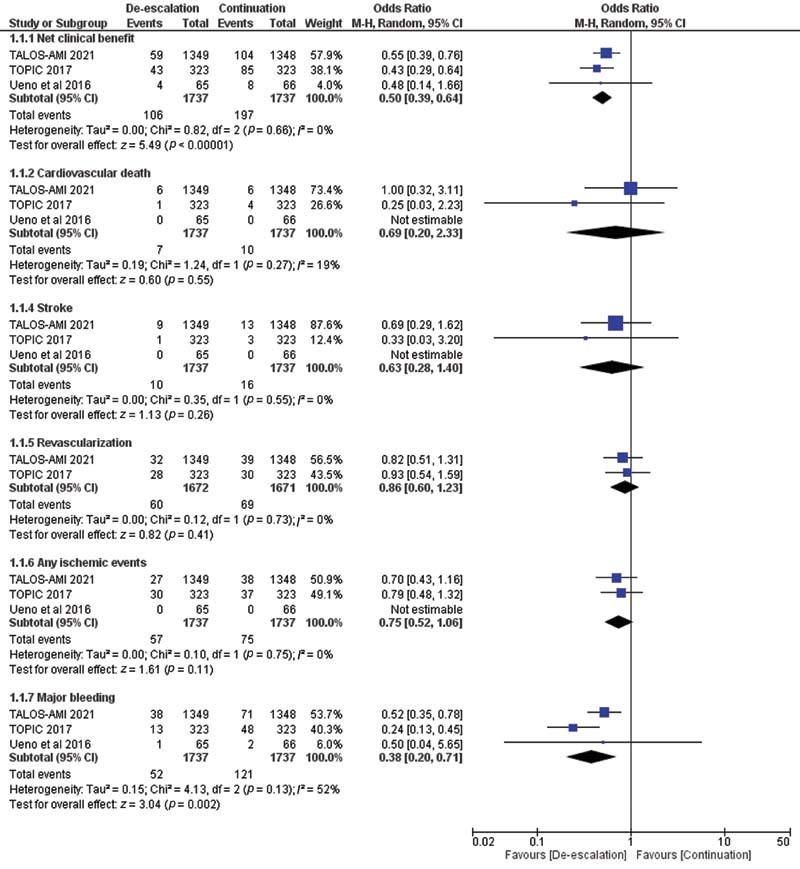
Forest plot of efficacy and safety outcomes. CI, confidence interval; M-H, Mantel-Haenszel.

Compared with the continuation strategy, deescalation strategy was associated with a significantly favorable net clinical benefit (OR = 0.50, 95% CI: 0.39, 0.64, *p* < 0.01, *I ^2^* = 0). The benefit was driven mainly by fewer bleeding events (OR = 0.38, 95% CI: 0.20, 0.71, *p* < 0.01, *I ^2^* = 52). There were no differences between groups in terms of cardiovascular deaths, strokes, revascularization, or any ischemic events (OR = 0.69; 95% CI: 0.20, 2.33, *p* = 0.55, *I ^2^* = 19; OR = 0.63; 95% CI: 0.28, 1.40, *p* = 0.26, *I ^2^* = 0; OR = 0.86; 95% CI: 0.60, 1.23, *p* = 0.41, *I ^2^* = 0; OR = 0.75; 95% CI: 0.52, 1.06, *p* = 0.11, *I ^2^* = 0, respectively; Fig. 1 ).

Our analysis showed that in patients with ACS post-PCI, unguided DAPT deescalation strategy was associated with a higher net clinical benefits in comparison to continuation strategy with more potent P2Y _12_ inhibitors.

Our results are consistent with current evidence. 3 4 The superiority of the potent P2Y _12_ inhibitors (ticagrelor and prasugrel) over (clopidogrel) has been demonstrated in ACS patients mainly in the acute phase, because of greater ischemic benefit, but there is an associated increased risk of bleeding after that. Therefore, superiority of (ticagrelor and prasugrel) beyond 30 days of cardiac events is questionable. 3 Consequently, deescalating to clopidogrel beyond the acute phase is more appealing to minimize the risk of bleeding and cost. On the other hand, guided (platelet function testing dependent) deescalation strategies have their own limitations. One strategy is deescalation to clopidogrel guided by genotype, 7 other strategy is to stop aspirin after 3 months and continue with potent P2Y _12_ inhibitor monotherapy for 12 months. 8 Those strategies despite being effective with reduced bleeding events, they require certain protocols and resource but lack the simplicity and potential cost saving of unguided deescalation at 1 month. Therefore, to reflect and guide clinical practice, we included only trials that implemented unguided deescalation of DAPT which is more feasible and pragmatic.

Noteworthy, specific considerations should be applied when dealing with specific population with certain risk profile, like east Asians. East Asians have different and specific genetic profile compared with Caucasians, with higher prevalence of certain genetic polymorphisms which might lead to different ischemic-bleeding risk profile. 9 This might be one of the reasons why earlier conclusions were favoring guided deescalation strategies, at least in certain populations. 10 Now after new evidence accumulated, and based on our study, we think that considering unguided DAPT deescalation is an attractive and reasonable approach, especially in certain situations like high bleeding risk patients or socioeconomic constrains.

Our study is the first to quantitatively summarize efficacy and safety of unguided deescalation of DPAT post-PCI, yet have several limitations. Some of the limitations are limited number of trials, lack of patient level data, and underrepresentation of females. Also, results were driven by one large trial (TALOS AMI). 4

In conclusion, among patients with ACS undergoing PCI, unguided deescalation strategy of potent P2Y _12_ inhibitors to clopidogrel was associated with favorable net clinical outcome, driven by lower bleeding events. Large and long-term RCTs are needed to compare the efficacy and safety of unguided deescalation versus guided deescalation strategy.
